# Taxonomy and phylogeny of *Pseudochaetosphaeronema* associated with rubber trees from Yunnan Province, China

**DOI:** 10.3897/mycokeys.128.182163

**Published:** 2026-02-17

**Authors:** Rui-Fang Xu, Samantha C. Karunarathna, Kevin D. Hyde, Pattana Kakumyan, Eric H.C. McKenzie, Abdallah M. Elgorban, Fu-Qiang Yu, Alanoud T. Alfagham, Chayanard Phukhamsakda, Saowaluck Tibpromma

**Affiliations:** 1 School of Science, Mae Fah Luang University, Chiang Rai, 57100, Thailand School of Science, Mae Fah Luang University Chiang Rai Thailand; 2 Center of Excellence in Fungal Research, Mae Fah Luang University, Chiang Rai, 57100, Thailand Center of Excellence in Fungal Research, Mae Fah Luang University Chiang Rai Thailand; 3 Center for Yunnan Plateau Biological Resources Protection and Utilization & Yunnan International Joint Laboratory of Fungal Sustainable Utilization in South and Southeast Asia, College of Biology and Food Engineering, Qujing Normal University, Qujing 655099, China College of Biology and Food Engineering, Qujing Normal University Qujing China; 4 Microbial Products and Innovations Research Group, Mae Fah Luang University, Chiang Rai, 57100, Thailand Kunming Institute of Botany, Chinese Academy of Sciences Kunming China; 5 Bioeconomy Science Institute, Private Bag 92170, Auckland, New Zealand Center of Excellence in Biotechnology Research (CEBR), King Saud University Riyadh Saudi Arabia; 6 Center of Excellence in Biotechnology Research (CEBR), King Saud University, P.O. Box 2455, Riyadh 11451, Saudi Arabia College of Science, King Saud University Riyadh Saudi Arabia; 7 Yunnan Key Laboratory for Fungal Diversity and Green Development & Yunnan International Joint Laboratory of Fungal Sustainable Utilization in South and Southeast Asia, Germplasm Bank of Wild Species, Kunming Institute of Botany, Chinese Academy of Sciences, Kunming 650201, China Department Microbial Drugs, Helmholtz Centre for Infection Research (HZI) Braunschweig Germany; 8 Department of Botany and Microbiology, College of Science, King Saud University, P.O. Box 2455, Riyadh 11451, Saudi Arabia Microbial Products and Innovations Research Group, Mae Fah Luang University Chiang Rai Thailand; 9 Department Microbial Drugs, Helmholtz Centre for Infection Research (HZI), Inhoffenstrasse 7, 38124, Braunschweig, Germany Bioeconomy Science Institute Auckland New Zealand

**Keywords:** 1 new species, Dothideomycetes, fungi, multi-locus phylogeny, Pleosporales, saprobes

## Abstract

Approximately 77% of *Pseudochaetosphaeronema* species have been reported from China and Thailand in terrestrial habitats. We collected and isolated fruiting bodies of ascomycetous fungi in Yunnan Province, China, from dead branches of *Hevea brasiliensis*. Integrating morphology with phylogenetic analyses of combined ITS, LSU, SSU, and *tef*1-α sequence data, the strains were identified as a new species (*Pseudochaetosphaeronema heveae*), two new host records (*P. baoshanense* and *P. chiangraiense*), and a new collection (*P. xishuangbannaense*). Descriptions, illustrations, phylogenetic analysis results, and morphological comparisons with allied taxa of the four *Pseudochaetosphaeronema* species are provided. These findings expand the known diversity and host associations of *Pseudochaetosphaeronema*. In addition, based on morphological characteristics and molecular phylogenetic data, *P. hongheense* is synonymized under *P. baoshanense*.

## Introduction

*Hevea brasiliensis* (Euphorbiaceae) is a tropical tree extensively cultivated in Southeast Asia as the primary source of natural rubber (Nair 2021). Rubber is one of Yunnan’s major cash crops, particularly in Xishuangbanna (China), where plantations have expanded rapidly over recent decades and now cover a significant portion of the landscape, driving local income (Ling et al. 2022). Research on fungi associated with *Hevea brasiliensis* has primarily focused on pathogenic fungi, with an emphasis on identifying and controlling leaf, stem, and root diseases (Lin et al. 2021). Endophytic fungi have also received attention, particularly regarding their potential roles in plant growth, stress tolerance, and biocontrol (Xu et al. 2019). More recently, attention has expanded to saprobic fungi, which decompose dead plant material (Senwanna et al. 2021; Nizamani et al. 2023). Numerous taxa have been described from this host, underscoring its role as a reservoir of fungal diversity (Theodoro and Batista 2014; Senwanna et al. 2021; Xu et al. 2022, 2023, 2024, 2025; Manawasinghe et al. 2024; Liu et al. 2025).

The family Macrodiplodiopsidaceae, with *Macrodiplodiopsis* as its type genus, was established by Crous et al. (2015) within the suborder Massarineae (Pleosporales, Dothideomycetes). Subsequent studies expanded its generic composition to include *Camarographium*, *Macrodiplodiopsis*, *Pseudochaetosphaeronema*, and *Pseudomonodictys* in this family (Ariyawansa et al. 2015; Tanaka et al. 2015). However, later phylogenetic analyses and taxonomic revisions demonstrated that some of these genera are placed in other families (Wanasinghe et al. 2017; Phookamsak et al. 2022). As a result, the current circumscription of Macrodiplodiopsidaceae is more restricted, comprising only *Macrodiplodiopsis* and *Pseudochaetosphaeronema* (Wijayawardene et al. 2022; Hyde et al. 2024). Members of the family are mainly saprobes on decaying plants, although some species have been reported as opportunistic human pathogens (e.g. *Pseudochaetosphaeronema larense*, *P. martinelli*) (Ahmed et al. 2014, 2015; Boonmee et al. 2021; De Silva et al. 2022). Macrodiplodiopsidaceae is characterized by dark-brown, obovoid, asymmetric, euseptate ascospores in the sexual morph and by globose to subglobose conidiomata with ellipsoid to clavate conidia in the asexual morph (Crous et al. 2015).

*Pseudochaetosphaeronema*, one of the genera in Macrodiplodiopsidaceae, was established by Punithalingam (1979) with *P. larense* as the type species and currently comprises 20 species in Species Fungorum (2026). Species of *Pseudochaetosphaeronema* exhibit a widespread but predominantly tropical and subtropical distribution, with the highest diversity reported from Asia, particularly in China (Yunnan Province), and Thailand (Tibpromma et al. 2018; Jayasiri et al. 2019; Hyde et al. 2020; Boonmee et al. 2021; De Silva et al. 2022; Manawasinghe et al. 2024; Xu et al. 2024; Apurillo et al. 2025; Du et al. 2025; Lu et al. 2025; Wanasinghe et al. 2025); additional records have been reported from Australia, Venezuela, and Martinique (Punithalingam 1979; Ahmed et al. 2015; Tan and Shivas 2024). The genus has a broad host range, occurring primarily as a saprobe on diverse woody plants, including *Bruguiera cylindrica*, *Cercis chinensis*, *Coffea* sp., *Hevea brasiliensis*, *Magnolia garrettii*, *Olea europaea*, and *Tamarindus* sp. (Boonmee et al. 2021; De Silva et al. 2022; Li et al. 2023; Manawasinghe et al. 2024; Xu et al. 2024; Apurillo et al. 2025; Du et al. 2025; Lu et al. 2025). The members of *Pseudochaetosphaeronema* have been isolated as endophytes from *Geijera salicifolia* and *Ginkgo biloba* (Zhang et al. 2016; Tan and Shivas 2024) and as an opportunistic pathogen reported from human feet (Punithalingam 1979; Ahmed et al. 2014, 2015). Morphologically, the asexual morph is characterized by black, obpyriform pycnidia with a long neck, hyaline conidiophores, monoblastic or monophialidic conidiogenous cells, and subspherical to ellipsoidal conidia (Apurillo et al. 2025; Lu et al. 2025), while the sexual morph produces black, globose to subglobose ascomata with bitunicate asci and hyaline, fusiform ascospores (Boonmee et al. 2021; Li et al. 2023; Xu et al. 2024; Lu et al. 2025).

In this study, four *Pseudochaetosphaeronema* species were collected and isolated from rubber plants. Based on morphology and multi-gene phylogenetic analyses, we introduce a new species, report two new host records, and document an additional collection of *Pseudochaetosphaeronema* from *H. brasiliensis* in Yunnan Province, China. In previous studies, three species of *Pseudochaetosphaeronema* (*P. lincangense*, *P. puerense*, and *P. xishuangbannaense*) were discovered on rubber trees. This study increases the number of *Pseudochaetosphaeronema* species associated with *H. brasiliensis* to six. In addition, based on our phylogenetic analyses, *P. hongheense* (KUNCC 23–16774 and KUNCC 25–19456) is grouped together with *P. baoshanense* strains; therefore, we synonymized it under *P. baoshanense* based on morphology and multi-locus phylogenetic analyses.

## Materials and methods

### Sampling, examination, and isolation

Dead decaying branches of *Hevea brasiliensis* with ascomycetous fungal fruiting bodies were collected from Yunnan Province in China. Ecological data were recorded in accordance with Rathnayaka et al. (2024). Samples were transported to the mycology laboratory of Qujing Normal University in sealed plastic bags for further processing. Macro-morphological characteristics of fungal fruiting bodies were examined using a stereomicroscope (Leica S8AP0, Tokyo, Japan), while micro-morphological features were observed and photographed with a compound microscope (Olympus BX53, Tokyo, Japan). Fungi were isolated using single-spore isolation, as described by Senanayake et al. (2020). All morphological features were measured using Tarosoft (R) Image Framework version 0.9.7. and photographic plates were edited and combined in Adobe Photoshop CC 2017. Herbarium specimens were deposited at the Guizhou Medical University (**GMB-W**), China. Living cultures are deposited in the Guizhou Medical University Culture Collection (**GMBCC**). Facesoffungi (FoF) numbers and Index Fungorum Registration Identifiers (IF) were obtained as per Jayasiri et al. (2015) and Index Fungorum (2026), respectively. The data is also deposited in the Greater Mekong Subregion database (Chaiwan et al. 2021).

### DNA extraction, PCR amplification, and sequencing

Fungal genomic DNA was extracted from one or two-month-old fresh fungal mycelium growing on potato dextrose agar (PDA) using a DNA Extraction Kit-BSC14S1 (BioFlux, Hangzhou, P.R. China) following the manufacturer’s instructions. The DNA product was kept at 4 °C for DNA amplification and at −20 °C for long-term preservation. DNA was amplified by polymerase chain reaction (PCR) for four genes: internal transcribed spacer region (ITS), partial 28S large subunit nuclear ribosomal DNA (LSU), partial 18S small subunit rDNA (SSU), and partial translation elongation factor 1-alpha (*tef*1-α). The partial ITS was amplified with ITS5 and ITS4 (White et al. 1990); LSU with LR0R and LR5 (Vilgalys and Hester 1990); SSU with NS1 and NS4 (White et al. 1990); and *tef*1-α with EF1-983F and EF1-2218R (Rehner and Buckley 2005). PCR was carried out in 25 μL reaction mixtures containing 12.5 μL 2× Master Mix (EasyTaq™ DNA Polymerase, dNTPs, and buffer; Beijing TransGen Biotech, Beijing, China), 8.5 μL ddH_2_O, 2 μL template DNA, and 1 μL of each primer (10 pM). The thermal cycling program followed Tibpromma et al. (2018) and Tian et al. (2024): initial denaturation at 94 °C for 3 min; 35 cycles of 94 °C for 30 s, annealing at 56 °C for 50 s, and extension at 72 °C for 1 min; and a final extension at 72 °C for 10 min. PCR products were purified and sequenced at Sangon Biotech Co., Kunming, China.

### Phylogenetic analyses

**Figure 1.**
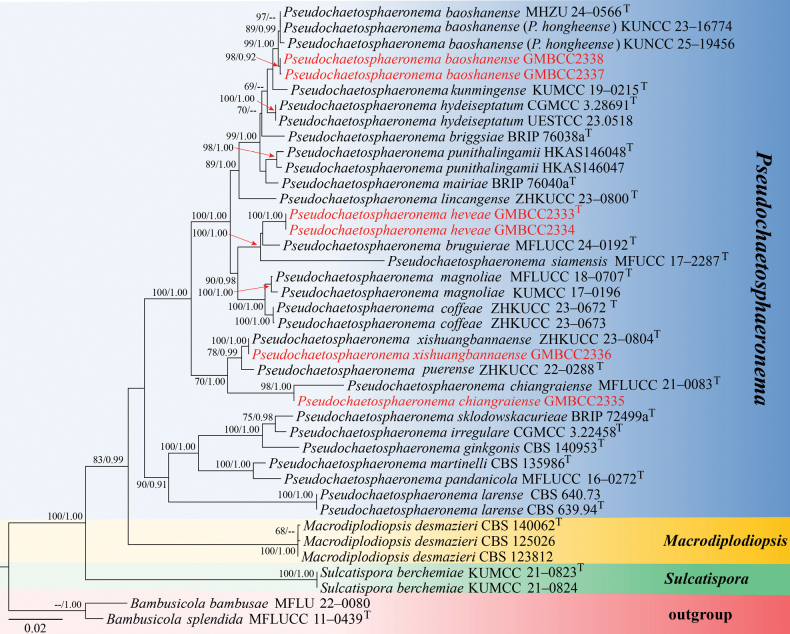
Phylogram generated from maximum likelihood analysis based on combined LSU, ITS, SSU, and *tef*1-α sequences of 40 strains. Bootstrap values (ML ≥ 60%) and Bayesian posterior probabilities (BPP ≥ 0.90) are shown at the nodes. The tree is rooted with *Bambusicola bambusae* (MFLU 22–0080) and *B. splendida* (MFLUCC 11–0439). New isolates are indicated in red; ex-type strains are marked with “T.” Bootstrap values (ML ≤ 60%) and Bayesian posterior probabilities (BYPP ≤ 0.90) are only shown at the nodes for new isolates of this study.

Newly generated sequences were subjected to BLAST searches against NCBI (https://blast.ncbi.nlm.nih.gov/Blast.cgi) to identify closely related taxa. Reference sequences from relevant publications (Du et al. 2025; Lu et al. 2025; Wanasinghe et al. 2025) and BLAST results were downloaded from GenBank and combined with the new sequences (Table 1). The relevant sequence data and newly generated DNA sequences were combined, and automatic alignment and editing were completed using OFPT (Zeng et al. 2023). Afterward, all datasets were concatenated into FASTA files; the alignments were visually checked and manually improved where necessary using the AliView program (Larsson 2014). Phylogenetic analyses were referenced in Dissanayake et al. (2020) and conducted using maximum likelihood (ML) and Bayesian posterior probability (BYPP) algorithms on the CIPRES Science Gateway portal (https://www.phylo.org/). ML analyses were performed with RAxML-HPC v.8, using the GTRGAMMA substitution model and 1,000 rapid bootstrap replicates. Bayesian analyses were conducted in MrBayes v.3.2.7a, with substitution models selected using MrModeltest v.2.2 (Nylander 2004); the best model for ITS, LSU, SSU, and *tef*1-α was GTR+I+G. Six simultaneous Markov chains were run for one million generations, automatically stopped when the topological convergence diagnostic reached 0.01, with a 25% burn-in. Trees were sampled every 100^th^ generation. The resulting trees were visualized using FigTree v.1.4.0 (Rambaut 2012) and edited in Microsoft PowerPoint 2021 and Adobe Photoshop CC 2017. The final alignments and trees were deposited in TreeBASE, under submission ID 32404 (Fig. 1) (http://www.treebase.org/).

**Table 1.** Taxa used in the phylogenetic analyses and their corresponding strain numbers and GenBank accession numbers. Sequences generated from this study are shown in bold; ex-type species strains are represented with T; sequence data that are not publicly available are mentioned with N/A.

Species	Strain numbers	GenBank Accession Numbers			
		ITS	LSU	SSU	*tef*1-α
*Bambusicola bambusae*	MFLU 22–0080	ON764309	ON764310	ON764313	KP761722
*Bambusicola splendida*	MFLUCC 11–0439 T	NR_121549	NG_058659	NG_063550	KP761726
*Macrodiplodiopsis desmazieri*	CBS 123812	KR873234	KR873269	N/A	N/A
*Macrodiplodiopsis desmazieri*	CBS 125026	KR873235	KR873270	N/A	N/A
*Macrodiplodiopsis desmazieri*	CBS 140062 T	KR873240	NG_058182	N/A	N/A
*Pseudochaetosphaeronema baoshanense*	MHZU 24–0566 T	PQ608323	PQ608156	PQ608010	PQ628168
*Pseudochaetosphaeronema baoshanense*	KUNCC 23–16774	PV742914	PV742970	PV743023	PV700649
*Pseudochaetosphaeronema baoshanense*	KUNCC 25–19456	PV742915	PV742971	PV743024	PV700650
***Pseudochaetosphaeronema baoshanense***	**GMBCC2337**	**PX651461**	**PX651455**	**PX651467**	**PX614647**
***Pseudochaetosphaeronema baoshanense***	**GMBCC2338**	**PX651462**	**PX651456**	**PX651468**	**PX614648**
*Pseudochaetosphaeronema briggsiae*	BRIP 76038a T	PQ431211	PQ431202	N/A	PQ724287
*Pseudochaetosphaeronema bruguierae*	MFLUCC 24–0515 T	PP989295	PP989290	PP989296	PQ273803
*Pseudochaetosphaeronema chiangraiense*	MFLUCC 21–0083 T	NR_175744	NG_081523	N/A	MZ476770
***Pseudochaetosphaeronema chiangraiense***	**GMBCC2335**	**PX651459**	**PX651453**	**PX651465**	**PX614646**
*Pseudochaetosphaeronema coffeae*	ZHKUCC 23–0672 T	PQ608321	PQ608154	PQ608008	PQ628166
*Pseudochaetosphaeronema coffeae*	ZHKUCC 23–0673	KU365986	KU365985	KU365983	KU365984
*Pseudochaetosphaeronema ginkgonis*	CBS 140953 T	KU365986	KU365985	KU365983	KU365984
***Pseudochaetosphaeronema heveae***	**GMBCC2333 T**	**PX651457**	**PX651451**	**PX651463**	**PX614644**
***Pseudochaetosphaeronema heveae***	**GMBCC2334**	**PX651458**	**PX651452**	**PX651464**	**PX614645**
*Pseudochaetosphaeronema hydeiseptatum*	CGMCC 3.28691 T	PQ773407	PQ773427	PQ773443	PV059187
*Pseudochaetosphaeronema hydeiseptatum*	UESTCC 23.0518	PQ773408	PQ773428	PQ773444	PV059188
*Pseudochaetosphaeronema irregulare*	CGMCC 3.22458 T	NR_191250	NG_243262	NG_242957	OQ809057
*Pseudochaetosphaeronema kunmingense*	KUMCC 19–0215 T	NR_170016	NG_075313	NG_070328	MN794017
*Pseudochaetosphaeronema larense*	CBS 639.94 T	KF015655	KF015610	KF015651	KF015683
*Pseudochaetosphaeronema larense*	CBS 640.73	NR_132038	NG_057978	NG_061147	KF015684
*Pseudochaetosphaeronema lincangense*	ZHKUCC 23–0800 T	OR853095	OR922336	OR922342	OR966290
*Pseudochaetosphaeronema magnoliae*	KUMCC 17–0196	OM212458	OL813498	OL824794	ON203110
*Pseudochaetosphaeronema magnoliae*	MFLUCC 18–0707 T	OM212457	OL813497	OL824793	ON203109
*Pseudochaetosphaeronema mairiae*	BRIP 76040a T	PQ431212	PQ431203	N/A	N/A
*Pseudochaetosphaeronema martinelli*	CBS 135986 T	NR_132930	NG_056290	NG_062412	KR909320
*Pseudochaetosphaeronema pandanicola*	MFLUCC 16–0272 T	MH275082	MH260316	MH260356	N/A
*Pseudochaetosphaeronema puerense*	ZHKUCC 22–0288 T	OR807846	OR807850	OR807848	OR966288
*Pseudochaetosphaeronema punithalingamii*	HKAS146048 T	PV742916	PV742972	PV743025	PV700651
*Pseudochaetosphaeronema punithalingamii*	HKAS146047	PV742917	PV742973	PV743026	PV700652
*Pseudochaetosphaeronema siamensis*	MFUCC 17–2287 T	MK347743	MK347960	MK347851	MK360074
*Pseudochaetosphaeronema sklodowskacurieae*	BRIP 72499a T	OP599632	OP598068	N/A	N/A
*Pseudochaetosphaeronema xishuangbannaense*	ZHKUCC 23–0804 T	OR853097	OR922338	OR922344	OR966286
*Pseudochaetosphaeronema xishuangbannaense*	ZHKUCC 23–0805	OR853098	OR922339	OR922345	OR966287
***Pseudochaetosphaeronema xishuangbannaense***	**GMBCC2336**	**PX651460**	**PX651454**	**PX651466**	**N/A**
*Sulcatispora berchemiae*	KUMCC 21–0823 T	ON009126	ON009110	ON009094	ON009269
*Sulcatispora berchemiae*	KUMCC 21–0824	ON009127	ON009111	ON009095	ON009270

## Results

### Phylogenetic analyses

The phylogenetic tree topologies obtained from RAxML and BI analyses were essentially similar. The RAxML analysis of the combined dataset yielded the best-scoring tree (Fig. 1), which was constructed from 3,167 base pairs (799 bp for LSU, 535 bp for ITS, 1,014 bp for SSU, and 819 bp for *tef*1-α). The final ML optimization likelihood value was -11667.153902. The matrix contained 650 distinct alignment patterns, with 11.78% of the characters being undetermined or gaps. The GTR+I+G model was applied for the combined ITS, LSU, SSU, and *tef*1-α dataset. The estimated base was: A = 0.239072, C = 0.251367, G = 0.268721, T = 0.240840; substitution rates AC = 1.455396, AG = 3.219017, AT = 1.80661, CG = 1.384182, CT = 8.948669, GT = 1.000000; and gamma distribution shape parameter *α* = 0.128109. The final RAxML tree is shown in Fig. 1.

In this phylogenetic tree, the results are similar to those reported by Du et al. (2025) and Wanasinghe et al. (2025). In this study, our six isolates clustered within *Pseudochaetosphaeronema*. Two strains of our isolates (GMBCC2337 and GMBCC2338) were grouped with *P. baoshanense* (MHZU 24–0566, holotype) and *P. hongheense* (KUNCC 23–16774: ex-type, KUNCC 25–19456) with 99% ML/1.00 BYPP statistical support. Another two of our strains (GMBCC2333, GMBCC2334) formed an independent lineage within *Pseudochaetosphaeronema* and were well distinct from the closely related species (*P. bruguierae*, MFLU 24–0192, holotype). The remaining two strains clustered with members of *Pseudochaetosphaeronema*. Strain GMBCC2335 clustered with the ex-type of *P. chiangraiense* (MFLUCC 21–0083) with 98% ML and 1.00 BYPP support, and GMBCC2336 clustered with the ex-type strain of *P. xishuangbannaense* (ZHKUCC 23–0804) with 100% ML and 1.00 BYPP support, which supports that they are the same species (*P. chiangraiense* and *P. xishuangbannaense*, respectively).

### Taxonomy

#### Pseudochaetosphaeronema baoshanense

Taxon classificationFungiPleosporalesMacrodiplodiopsidaceae

L. Lu, K.D. Hyde & Tibpromma Fungal Diversity 25: 1–275 (2025)

281EAF88-03D3-5D1B-9C73-90AF400ABA2E

Index Fungorum: IF903641

Facesoffungi Number: FoF17573

Fig. 2

##### Description.

***Saprobic*** on a decaying branch of *Hevea brasiliensis*. **Sexual morph**: Undetermined. **Asexual morph**: Coelomycetous. ***Conidiomata*** 140–170 × 170–200 μm (x̄ = 153 × 184 μm, n = 10), solitary, globose to subglobose or pyriform, dark brown, immersed or erumpent, with black spots on the host surface, unilocular. ***Conidiomatal wall*** 50–60 μm wide, composed of two layers, outer layers brown, inner layers pale brown to hyaline, arranged in a *textura angularis* to *textura prismatica*, darker at the outer layer, fusing and indistinguishable from the host tissues. ***Conidiophores*** arising from the inner layers lining the conidioma, or at the base, often reduced to conidiogenous cells. ***Conidiogenous cells*** 6–10 × 2–3 μm (x̄ = 7.23 × 2.14 μm, n = 15), monophialidic, cylindrical, integrated, hyaline or pale brown, smooth-walled. ***Conidia*** 11–15 × 5–6 μm (x̄ = 12.4 × 5.6 μm, n = 40), oblong to ellipsoidal, or fusoid, with rounded ends, sometimes truncate and narrow at the base, yellowish to pale brown, smooth, thick-walled, 1–3-septate, some slightly constricted at the septum.

**Figure 2.**
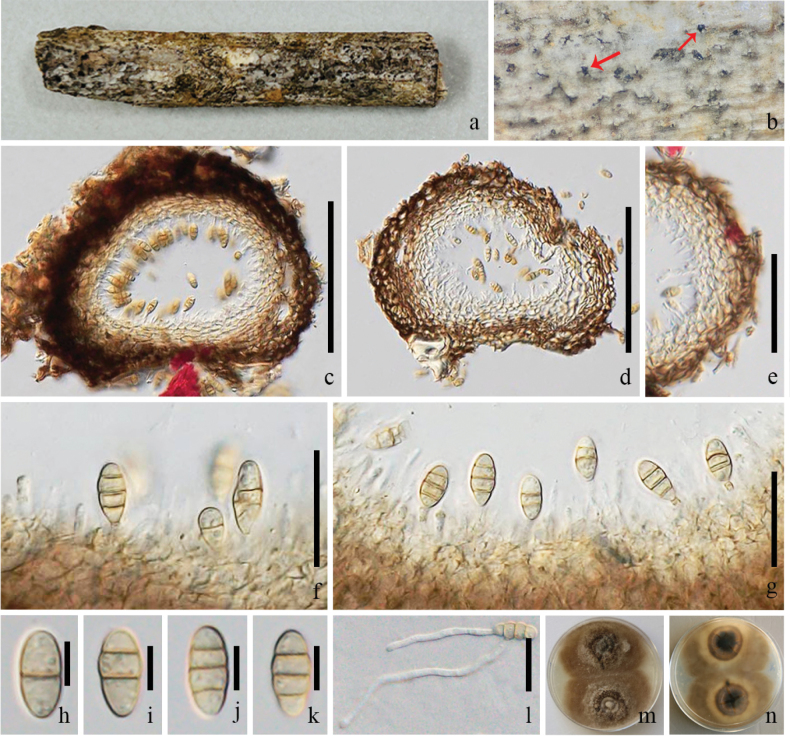
*Pseudochaetosphaeronema baoshanense* (GMB-W1228, New host record). **a**. Host branch piece; **b**. Conidiomata on host (arrows show conidiomata); **c, d**. Longitudinal section of conidiomata; **e**. Conidiomatal wall; **f, g**. Conidiogenous cells with conidia; **h–k**. Conidia; **l**. Germinated conidium; **m, n**. Colonies on PDA (surface and reverse). Scale bars: 100 μm (**c, d**); 50 μm (**e**); 20 μm (**f, g, l**); 5 μm (**h–k**).

##### Culture characteristics.

Conidia germinated on PDA within 12 hours. Colonies circular, raised, brown, with entire margin; reverse dark brown at the center, fading to pale brown at the edge.

##### Material examined.

China, • Yunnan Province, Dehong Dai and Jingpo Autonomous Prefecture, Mang City, on a decaying branch of *Hevea brasiliensis*, 18 April 2024, Rui-Fang Xu, DHR23 (GMB-W1227), living culture GMBCC2337; China, Yunnan Province, Lincang City, 28 July 2022, LCR24 (GMB-W1228), living culture GMBCC2338.

##### Known distributions.

China, Yunnan Province (Lu et al. 2025; Wanasinghe et al. 2025; this study).

##### Known hosts.

*Coffea* sp. (Lu et al. 2025), dead twigs of an unknown deciduous plant (Wanasinghe et al. 2025), *Hevea brasiliensis* (this study).

##### Notes.

Phylogenetic analyses show that strains GMBCC2337 and GMBCC2338 clustered with *Pseudochaetosphaeronema baoshanense* (MHZU 24–0566, holotype) and *P. hongheense* (KUNCC 23–16774; KUNCC 25–19456) with 99% ML and 1.00 BI support (Fig. 1). Morphologically, the species isolated from *Hevea brasiliensis* is similar to the species described by Lu et al. (2025) from *Coffea* sp., in having cylindrical conidiogenous cells and yellowish to brown, oblong to ellipsoidal or fusoid conidia. However, the conidiomata wall of our samples (GMB-W1227, GMB-W1228) is thicker than the holotype of *P. baoshanense* (MHZU 24–0566) (50–60 μm vs. 20–40 μm), and conidia of our samples are sometimes slightly constricted at the septa, whereas in the holotype they are non-constricted. While the ITS, LSU, and SSU regions were identical between the new isolates (GMBCC2337 and GMBCC2337) and *P. baoshanense* (MHZU 24–0566), only the *tef*1-α gene region showed four base pairs differences. Based on the combined molecular evidence, our collections are identified as *P. baoshanense*. According to our knowledge, this is the first report of *P. baoshanense* on *Hevea brasiliensis*.

*Pseudochaetosphaeronema hongheense* was introduced by Wanasinghe et al. (2025), and this fungus was isolated from Honghe, Yunnan Province. In the present study, *P. hongheense* (KUNCC 23–16774, KUNCC 25–19456) clusters with *P. baoshanense* (MHZU 24–0566, holotype) with 97% ML support, but Bayesian support is low. Pairwise nucleotide comparisons showed no differences in ITS, LSU, and *tef*1-α sequences, with only a base-pair gap in SSU. Morphologically, *P. hongheense* (HKAS146045, holotype) and *P. baoshanense* (MHZU 24–0566, holotype) are similar in conidiomatal structure, conidiogenous cells, and brown, septate conidia. However, *P. baoshanense* differs from *P. hongheense* in thicker conidiomatal wall (20–40 μm vs. 5–15 μm), conidia that are oblong to ellipsoidal or fusoid and not constricted at the septa, and in producing 1–3-septate conidia that are only slightly thick-walled at maturity (Lu et al. 2025). *Pseudochaetosphaeronema hongheense* is characterized by distinctly constricted, consistently 3-septate, thick-walled conidia (Wanasinghe et al. 2025). Based on the molecular and morphological evidence, *P. hongheense* is considered a synonym of *P. baoshanense*.

#### Pseudochaetosphaeronema chiangraiense

Taxon classificationFungiPleosporalesMacrodiplodiopsidaceae

Wijesinghe, Boonmee & K.D. Hyde, Fungal Diversity 111: 75 (2021)

A12C9031-F077-5A8A-A05C-D85ABDCB7425

Index Fungorum: IF558549

Facesoffungi Number: FoF09950

Fig. 3

##### Description.

***Saprobic*** on a decaying branch of *Hevea brasiliensis*. **Sexual morph: *Ascomata*** 170–280 × 200–280 μm (x̄ = 221 × 223 μm, n = 10), solitary, scattered, immersed, uniloculate, globose to subglobose. ***Peridium*** 30–50 μm wide, composed of several brown to pale brown cells of *textura angularis*. ***Hamathecium*** 1–2 μm wide, numerous, filiform, unbranched, pseudoparaphyses. ***Asci*** 80–123 × 23–29 μm (x̄ = 99 × 25 μm, n = 15), 8-spored, bitunicate, fissitunicate, cylindrical to clavate, with a short pedicel, apex rounded, with ocular chamber. ***Ascospores*** 20–32 × 7–13 μm (x̄ = 26 × 10 μm, n = 30), overlapping, 2–3 seriate, fusiform, with obtuse ends, 1-septate at the center, constricted at the septa, hyaline, guttulate, thick and smooth-walled. **Asexual morph**: Undetermined.

**Figure 3.**
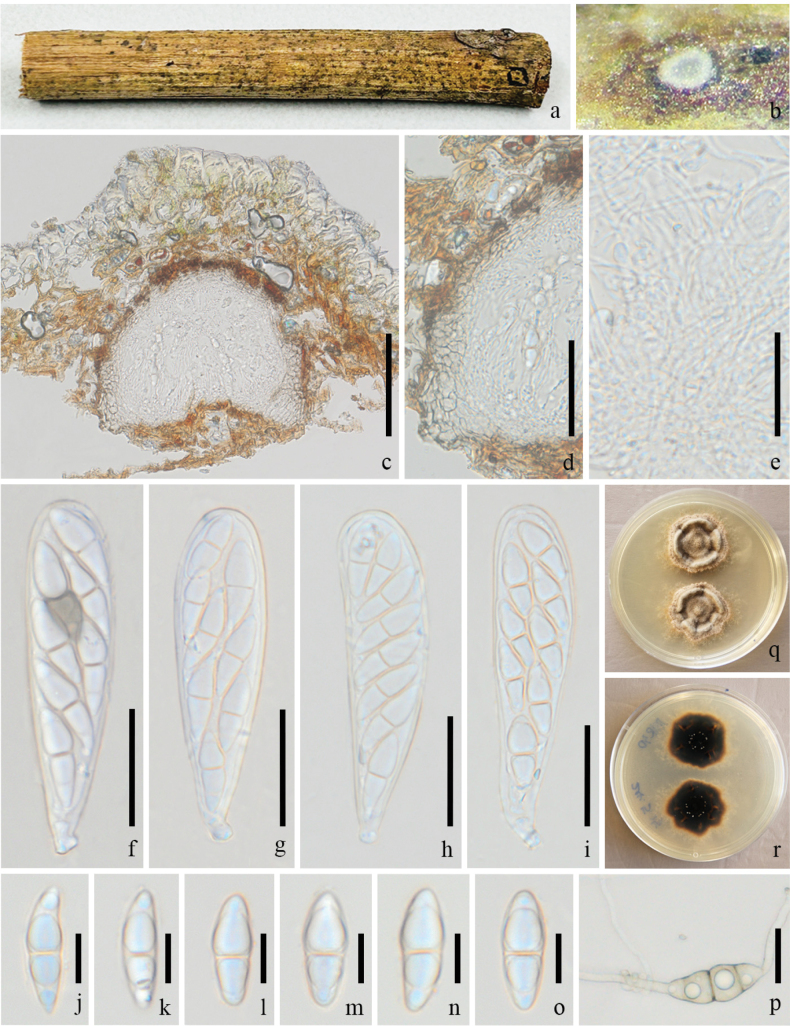
*Pseudochaetosphaeronema chiangraiense* (GMB-W1225, New host record). **a**. Host branch piece; **b**. Ascoma on the host; **c**. Longitudinal section of ascoma; **d**. Partial peridium; **e**. Pseudoparaphyses; **f–i**. Asci; **j–o**. Ascospores; **p**. Germinated ascospore; **q, r**. Colonies on PDA (surface and reverse). Scale bars: 100 μm (**c**); 30 μm (**d–k**); 20 μm (**l–p**).

##### Culture characteristics.

Ascospores germinated on PDA within 24 hours, and ascospores turned brown after germination. Colonies irregular, wrinkled, brown, crateriform with undulate margin; reverse dark brown.

##### Material examined.

China, • Yunnan Province, Dehong Dai and Jingpo Autonomous Prefecture, Mang City, on a decaying branch of *Hevea brasiliensis*, 18 April 2024, Rui-Fang Xu, DHR30 (GMB-W1225), living culture GMBCC2335.

##### Known distributions.

Thailand (Boonmee et al. 2021) and Yunnan Province, China (Li et al. 2023; this study).

##### Known hosts.

*Tamarindus* sp. (Boonmee et al. 2021), *Olea europaea* (Li et al. 2023), and *Hevea brasiliensis* (this study).

##### Notes.

*Pseudochaetosphaeronema chiangraiense* was collected from *Tamarindus* sp. in Chiang Rai, Thailand (Boonmee et al. 2021), and was also later recorded on *Olea europaea* in Sichuan Province, China (Li et al. 2023). In this study, our isolate (GMBCC2335) was grouped with *P. chiangraiense* (MFLUCC 21–0083, ex-type). The morphology of our collection (GMB-W1225) is similar to the holotype of *P. chiangraiense* (Boonmee et al. 2021), but differs only in size: holotype with a thinner peridium when compared to our collection (13–17 μm vs. 30–50 μm), and a bit big in pseudoparaphyses (2–4.5 μm vs. 1–2 μm); larger asci (50–110 × 15–30 μm vs. 80–123 × 23–29 μm), and longer ascospores (20–45 μm vs. 20–32 μm). However, the new isolate was genetically identical to *P. chiangraiense* in the ITS, LSU, and SSU regions but differed by three base pairs in the *tef*1-α gene. Therefore, we identify our strain as *P. chiangraiense* based on phylogenetic and morphological evidence. According to our knowledge, this is the first record of *P. chiangraiense* on *H. brasiliensis* in China.

#### Pseudochaetosphaeronema heveae

Taxon classificationFungiPleosporalesMacrodiplodiopsidaceae

R.F. Xu, K.D. Hyde & Tibpromma sp. nov.

D18B6048-16B5-545F-8D03-71D0B8F3FABD

Index Fungorum: IF904508

Facesoffungi Number: FoF18921

Fig. 4

##### Etymology.

Refers to the host genus *Hevea*.

##### Holotype.

GMB-W1222

##### Description.

***Saprobic*** on a decaying branch of *Hevea brasiliensis*. **Sexual morph**: Undetermined. **Asexual morph**: Coelomycetous. ***Conidiomata*** 125–170 × 140–225 μm (x̄ = 148 × 190 μm, n = 10), solitary to aggregated, globose to subglobose or pyriform, immersed to semi-immersed, unilocular, dark brown to black, appearing as black dots on the host surface. ***Conidiomatal wall*** 20–70 μm wide, brown to dark brown, *textura angularis*. ***Conidiophores*** reduced to conidiogenous cells. ***Conidiogenous cells*** 3–8 × 2–6 μm (x̄ = 6 × 4.4 μm, n = 10), monophialidic, cylindrical or ampulliform, hyaline, smooth-walled, with guttules. ***Conidia*** 8–14 × 3–4 μm (x̄ = 11.25 × 3.23 μm, n = 50), fusiform or fusoid, slightly curved, 1–2-septate, constricted at septum, cell swollen sometimes, rounded at both ends, hyaline, smooth, thin-walled, without a mucilaginous sheath.

**Figure 4.**
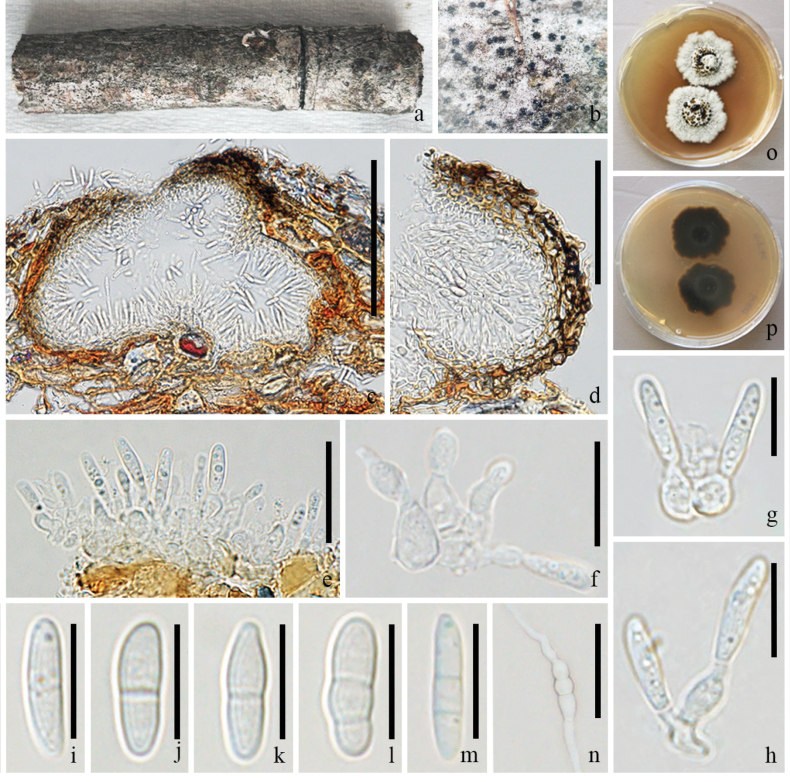
*Pseudochaetosphaeronema heveae* (GMB-W1222, Holotype). **a**. Host branch piece; **b**. Conidiomata on host; **c**. Longitudinal section of conidioma; **d**. Conidiomatal wall; **e–h**. Conidiogenous cells with conidia; **i–m**. Conidia; **n**. Germinated conidium; **o, p**. Colonies on PDA (surface and reverse). Scale bars: 100 μm (**c**), 50 μm (**d**), 30 μm (**e**), 10 μm (**f–n**).

##### Culture characteristics.

Conidia germinating on PDA within 12 hours. Colonies irregular, umbonate, white to cream, with a central brown droplet, undulate margin; reverse dark brown with pale brown edge, producing brown pigment on PDA media.

##### Material examined.

China, • Yunnan Province, Dehong Dai and Jingpo Autonomous Prefecture, Mang City, on a decaying branch of *Hevea brasiliensis*, 18 April 2024, Rui-Fang Xu, DHR27 (GMB-W1222, holotype), ex-type living culture GMBCC2333 = GMBCC2334.

##### Notes.

In the phylogenetic analyses, GMBCC2333 and GMBCC2334 formed a distinct lineage, sister to *P. siamensis* (MFLUCC 17–2287, ex-type) and *P. bruguierae* (MFLUCC 24–0515, ex-type) (Fig. 1). Sequence comparisons showed that GMBCC2333 differs from *P. siamensis* by 13/510 bp (ITS, 2.55%), 3/810 bp (LSU, 0.20%), 2/1,005 bp (SSU, 0.19%), and 99/645 bp (*tef*1-α, 15.35%); differences with *P. bruguierae* included 8/497 bp (ITS, 1.61%), 2/810 bp (LSU, 0.25%), and 28/893 bp (*tef*1-α, 3.14%). Morphologically, *P. heveae* (GMB-W1222, holotype) can be distinguished from *P. coffeae* (MHZU 23–0081, holotype) by smaller conidiomata (125–170 × 140–225 μm vs. 150–190 × 180–240 μm) and conidiogenous cells (3–8 × 2–6 μm vs. 6–9 × 3–6 μm), as well as shorter conidia (8–14 × 3–4 μm, 1–2-septate vs. 12–16 × 3–5 μm, 1–3-septate) (Lu et al. 2025). It also differs from *P. bruguierae* (MFLU 24–0192, holotype), which has larger conidiomata (230–400 × 300–370 μm), narrower conidiogenous cells (7–10 × 1–2.7 μm), and smaller conidia (5–10 × 2–3 μm), 2–3-septate with guttules (Apurillo et al. 2025). Based on molecular and morphological evidence, we introduce our new collection as a new species, *P. heveae*.

#### Pseudochaetosphaeronema xishuangbannaense

Taxon classificationFungiPleosporalesMacrodiplodiopsidaceae

R.F. Xu & Tibpromma, MycoKeys 103: 71–95 (2024) [as ‘ xishuangbannaensis’]

685CFB43-F334-588A-B627-227D7D617B8C

Index Fungorum: IF901422

Facesoffungi Number: FoF15198

Fig. 5

##### Description.

***Saprobic*** on a decaying branch of *Hevea brasiliensis*. **Sexual morph: *Ascomata*** 110–330 × 212–335 μm, (x̄ = 218 × 263 μm, n = 10), white spot on the surface, sometimes inconspicuous on host surface, solitary, scattered, immersed, globose to subglobose or pyiform, or ellipsoid, uni-loculate, brown, with ostiole. ***Ostiole*** 70–155 × 60–85 μm, (x̄ = 105 × 70 μm, n = 5), central, brown, papillate. ***Peridium*** 30–60 μm wide, composed of several layers, brown to pale brown to hyaline cells of *textura intricata*. ***Hamathecium*** comprises 2–3 μm wide, numerous, filiform, unbranched, hyaline, cellular pseudoparaphyses. ***Asci*** 80–110 × 16–30 μm (x̄ = 95 × 21 μm, n = 15), 8-spored, bitunicate, obovoid, short, distinct pedicel, apex rounded with an ocular chamber. ***Ascospores*** 26–36 × 10–12 μm (x̄ = 30 × 11 μm, n = 20), hyaline, fusiform, with pointed ends, 1-septate, occasionally 3-septate, constricted at the middle septum, wider upper cell, constricted at the septa, guttulate, thick-walled, with a thin mucilaginous sheath. **Asexual morph**: Undetermined.

**Figure 5.**
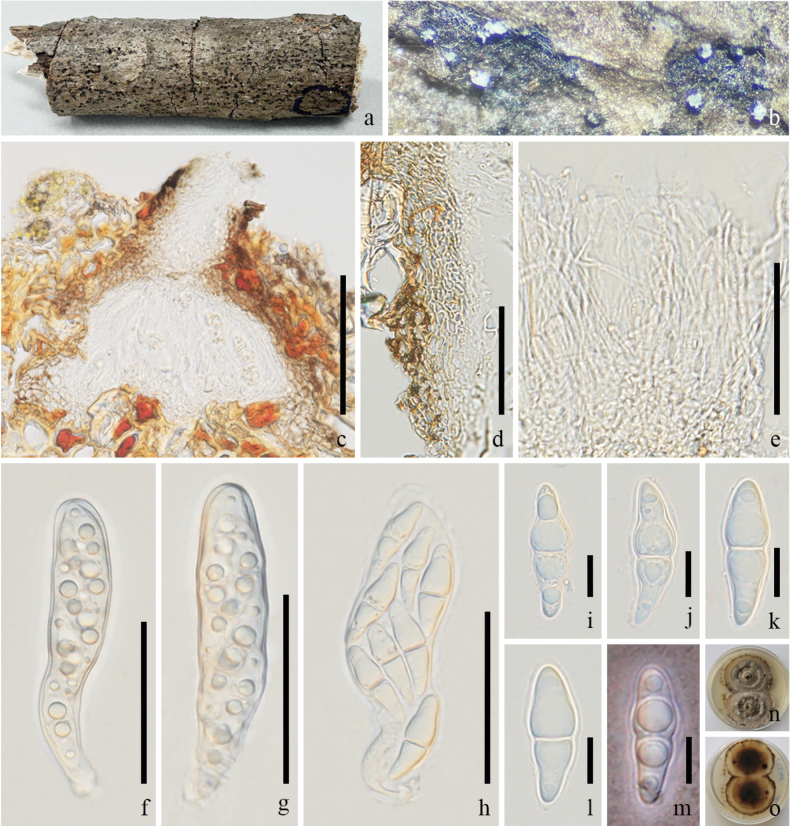
*Pseudochaetosphaeronema xishuangbannaense* (GMB-W1226). **a**. Host branch piece; **b**. Ascomata on the host; **c**. Longitudinal section of ascoma; **d**. Partial peridium; **e**. Pseudoparaphyses; **f–h**. Asci; **i–l**. Ascospores; **m**. Ascospore with India ink; **n, o**. Colonies on PDA (surface and reverse). Scale bars: 100 μm (**c**); 50 μm (**d–h**); 10 μm (**i–m**).

##### Culture characteristics.

Ascospores germinate on PDA within 24 hours. Colonies circular, umbonate, entire margin; brown from above, dark brown at center and edge, with brown ring on reverse.

##### Material examined.

China, • Yunnan Province, Dehong Dai and Jingpo Autonomous Prefecture, Mang City, on a decaying branch of *Hevea brasiliensis*, 18 April 2024, Rui-Fang Xu, DHR42 (GMB-W1226), living culture GMBCC2336.

##### Known distributions.

China, Yunnan Province, Xishuangbanna Dai Autonomous Prefecture (Xu et al. 2024), China, Yunnan Province, Dehong Dai and Jingpo Autonomous Prefecture (this study).

##### Known hosts.

*Hevea brasiliensis* (Xu et al. 2024, this study)

##### Notes.

In the phylogenetic analyses, strain GMBCC2336 formed a clade with strains of *P. xishuangbannaense* (ZHKUCC 23–0804 and ZHKUCC 23–0805) with 100% ML/1.00 BYPP statistical supports (Fig. 1). Compared with the holotype of *P. xishuangbannaense* (ZHKU 23–0107) with our sample (GMB-W1226) has smaller ascomata (110–330 × 212–335 μm vs. 270–410 × 370–480 μm), a thinner peridium (30–60 μm vs. 40–90 μm), smaller asci (80–110 × 16–30 μm vs. 130–180 × 25–35 μm), and smaller, 1–3-septate ascospores (26–36 × 10–12 μm vs. 30–50 × 10–20 μm, 3–5-septate) (Xu et al. 2024). Despite these size differences, the overall morphology and phylogeny align with those of *P. xishuangbannaense*. The nucleotide comparisons show differences of 3 bp (570 bp) and 1 bp (954 bp) of ITS and SSU, respectively, between the new isolate (GMBCC2336) and the ex-type of *P. xishuangbannaense* (ZHKUCC 23–0804). Therefore, strain GMBCC2336 is identified as *P. xishuangbannaense* and represents a newly collected specimen from Dehong, about 415 km from the type locality in Xishuangbanna.

## Discussion

This study provides new insights into the diversity of *Pseudochaetosphaeronema* associated with *Hevea brasiliensis* in Yunnan Province, China. We introduce *P. heveae* as a novel species, establish new host records for *P. baoshanense* and *P. chiangraiense*, and provide an additional collection of *P. xishuangbannaense*. The presence of multiple *Pseudochaetosphaeronema* taxa on a single host highlights the rich diversity of saprobes in tropical and subtropical ecosystems. The rubber tree hosts a wide variety of fungi due to the warm, humid climate of tropical and subtropical regions where it grows, which further promotes fungal development and diversity (Talley et al. 2002; Senwanna et al. 2021). The number of *Pseudochaetosphaeronema* species from *H. brasiliensis* now increases to six with the addition of *P. lincangense* and *P. puerense* by Manawasinghe et al. (2024) and Xu et al. (2024).

Moreover, based on multi-locus phylogenetic analyses, pairwise nucleotide comparisons, and morphology comparisons, *P. hongheense* is synonymized with *P. baoshanense*. We observed slight morphological differences among collections of *P. baoshanense*, even though they are from Yunnan Province; these differences may be due to their local environment (Baoshan (type locality), Honghe (Wanasinghe et al. 2025), Lincang, and Dehong (this study), host plant (coffee plant, dead twigs of an unknown deciduous tree, and rubber tree), or geographical origin.

The recognition of *Pseudochaetosphaeronema heveae* as a distinct new species is strongly supported by a combination of morphological and phylogenetic evidence. Although phylogenetic analyses placed *P. heveae* in a separate lineage from *P. siamensis*, the statistical support was relatively low. This limited resolution is likely due to insufficient information obtained with only ITS, LSU, and SSU markers within the genus, as observed in previous studies; by contrast, the present study, which included *tef*1-α, provided greater discriminatory power. Nonetheless, the consistent tree topologies recovered from both ML and BI analyses, coupled with stable morphological characters, lend confidence to the delimitation of *P. heveae*. This case highlights the importance of integrating multiple loci, especially protein-coding, in fungal taxonomy. The low phylogenetic support for *P. heveae* suggests that additional loci, such as *RPB2* and *TUB2*, may be needed to resolve intrageneric relationships among species. Our study was conducted extensively across Yunnan Province, and the high diversity of *Pseudochaetosphaeronema* species observed suggests that sampling of *Hevea brasiliensis* across Southeast Asia and South America may reveal more species (Boonmee et al. 2021; De Silva et al. 2022; Li et al. 2023; Xu et al. 2024; Lu et al. 2025). Future research integrating morphology, multigene phylogenetics, and ecological data will be crucial for clarifying species boundaries, host specificity, and functional roles of this genus in tropical ecosystems.

The discovery of *Pseudochaetosphaeronema baoshanense* and *P. chiangraiense* on *Hevea brasiliensis* represents new host records, reflecting the ecological adaptability of *Pseudochaetosphaeronema*. *Pseudochaetosphaeronema baoshanense* was originally described from *Coffea* sp. in Yunnan Province, China, while *P. chiangraiense* was first reported from *Tamarindus* sp. in Thailand and subsequently on *Olea europaea* in Sichuan Province, China (Boonmee et al. 2021; Li et al. 2023; Lu et al. 2025). Based on the distribution of their host plants across tropical and subtropical regions, *P. baoshanense* and *P. chiangraiense* appear well adapted to warm, humid environments. Their occurrence in these climatic conditions suggests that temperature plays an important role in shaping their ecological distribution and host ranges. Our study extends their host range to *Hevea*, suggesting that these fungi may be saprobes capable of colonizing a diverse range of woody substrates. Whether these species exhibit host preference or are generalist colonizers remains uncertain, but their repeated detection across unrelated hosts highlights the need for broader ecological surveys.

Our additional collection of *Pseudochaetosphaeronema xishuangbannaense* confirms its occurrences on *Hevea brasiliensis* beyond the original locality in Xishuangbanna, Yunnan (Xu et al. 2024). The isolate exhibited species-level consistency with the type material. Although some differences were observed in ascomata, peridium, asci, and ascospore size, such intraspecific variation is not uncommon among coelomycetous fungi; environmental or host-related factors may influence it. Further collections of *P. xishuangbannaense* from *Hevea* are needed to confirm its saprobic preferences in local rubber plantations. The species could potentially play a crucial role in wood decomposition.

Rubber tree plantations are managed forests where a single tree species is grown (Wang and Zhang 2025). However, some rubber plantations in China (in the provinces of Hainan and Yunnan) incorporate coffee, tea, and pineapple as intercrops (Qi et al. 2024). Among microorganisms, fungi are among the most important because they help break down material, recycle nutrients, and decompose dead material. This keeps the plantation ecosystem healthy. In this study, we found several *Pseudochaetosphaeronema* species on dead rubber tree wood in a saprobic life mode. However, they might switch their lifestyle. First, they live as endophytes within a healthy tree, and then become active decomposers when the wood dies. Furthermore, these wood-decaying fungi likely interact with other microbes.

In conclusion, this study documents the discovery of a new species, two new host records, and a new collection of *Pseudochaetosphaeronema* from *H. brasiliensis* in China. These findings not only expand the known diversity and host range of the genus but also underscore the ecological significance of saprobic fungi in rubber plantations. By integrating molecular and morphological data, we contribute to the taxonomic framework for *Pseudochaetosphaeronema* and lay a foundation for future studies on its biodiversity, ecology, and potential interactions with economically important hosts. Beyond documenting taxonomic novelties, this study emphasizes the broader ecological relevance of saprobic fungi associated with *H. brasiliensis*. A deeper understanding of these rubber-associated fungal communities will contribute to a more comprehensive assessment of plantation ecosystem health and provide a foundation for future studies on microbial interactions, functional diversity, and potential applications in biological control and biodegradation.

## Supplementary Material

XML Treatment for Pseudochaetosphaeronema baoshanense

XML Treatment for Pseudochaetosphaeronema chiangraiense

XML Treatment for Pseudochaetosphaeronema heveae

XML Treatment for Pseudochaetosphaeronema xishuangbannaense

## Additional information

### Use of AI

No use of AI was reported.

### Author ORCIDs

Rui-Fang Xu https://orcid.org/0000-0003-1207-8254

Samantha C. Karunarathna https://orcid.org/0000-0001-7080-0781

Kevin D. Hyde https://orcid.org/0000-0002-2191-0762

Pattana Kakumyan https://orcid.org/0000-0001-8281-1082

Eric H.C. McKenzie https://orcid.org/0000-0001-9400-7112

Abdallah M. Elgorban https://orcid.org/0000-0003-3664-7853

Fu-Qiang Yu https://orcid.org/0000-0001-7931-080X

Alanoud T. Alfagham https://orcid.org/0000-0001-9008-3679

Chayanard Phukhamsakda https://orcid.org/0000-0002-1033-937X

Saowaluck Tibpromma https://orcid.org/0000-0002-4706-6547

### Data availability

All of the data that support the findings of this study are available in the main text.
